# Getting clues from nature: the impact of grass hay on suckling piglets’ gastrointestinal growth and colonic microbiota

**DOI:** 10.3389/fcimb.2023.1341147

**Published:** 2024-01-10

**Authors:** Renjie Yao, An Cools, Hubèrt M. J. van Hees, Koen Chiers, Awot Teklu Mebratu, Marijke Aluwé, Dominiek Maes, Geert P. J. Janssens

**Affiliations:** ^1^ Department of Veterinary and Biosciences, Ghent University, Merelbeke, Belgium; ^2^ Department of Internal Medicine, Reproduction and Population Medicine, Ghent University, Merelbeke, Belgium; ^3^ Trouw Nutrition Research & Development, Amersfoort, Netherlands; ^4^ Department of Pathology, Ghent University, Merelbeke, Belgium; ^5^ Flanders Research Institute for Agriculture, Fisheries and Food (ILVO), Melle, Belgium

**Keywords:** grass hay, suckling piglets, gastrointestinal development, insoluble fibre, creep feed

## Abstract

**Introduction:**

The effect of dietary fiber on pig production has been extensively evaluated. Inspired by observations of the diet of wild, young piglets, this study aimed to examine the possibility of feeding grass hay to suckling piglets besides concentrated creep feed.

**Methods:**

The sow-nursed piglets in this study were divided into two groups based on balanced sow parities. The control group (CON, *n* = 7 sows) only received a regular, concentrated creep feed, while the treatment piglets (GH, *n* = 8 sows) were also provided with chopped grass hay from 2 days of age until weaning (28 days). At weaning, one piglet with a median weight was selected from each litter for post-mortem evaluation. Subsequently, six pigs around median weight per sow were grouped into nursery pens and monitored for their feed intake and body weight gain until 9 weeks of age.

**Results and discussion:**

Piglets in GH consumed, on average, 57 g of grass hay per piglet during the entire lactation period. The emptied weight of the small and large intestine was significantly greater in GH (280 vs. 228 g, 88.8 vs. 79.3 g, respectively, *p* < 0.05), and the length of the large intestine was stimulated by the grass hay (164 vs. 150 cm, *p* < 0.05). Morphologically, the villus height in the jejunum was higher in GH (*p* < 0.05). In the large intestine, the crypt depth of the mid-colon was lower in GH. Moreover, the short-chain fatty acid (SCFA) concentrations in the cecum were increased in GH compared to CON (1,179 vs. 948 µmol/g dry matter, *p* < 0.05), whereas in the colon, SCFA concentrations were lower in CON (341 vs. 278 µmol/g dry matter, *p* < 0.05). There was no major impact of grass hay inclusion on the colonic microbiota composition. Only a trend was observed for a lower inverse of the classical Simpson (InvSimpon) index and a higher abundance of *Lactobacillus* genera in GH. After weaning, no significant differences in feed intake and body weight gain were observed. In conclusion, supplementing the grass hay to suckling piglets led to alterations in intestinal morphology, increased SCFA fermentation in proximal sections of large intestine, stimulation of gastrointestinal tract growth, and subtle modifications in colonic microbiota.

## 1 Introduction

The weaning transition is a challenge in swine breeding husbandry. To maximize profits, early-weaning strategies at 3–4 weeks of age have been widely adopted on commercial farms. Such sudden transition of diets predisposes piglets to diarrhea, aberrant behaviors, growth stasis, and even more detriments associated with weaning stressors, eventually leading to production loss (Lallès et al., 2007; Summers et al., 2019). To address these concerns, creep feed is provided to familiarize the piglets during the suckling period with the solid feed after weaning and partly release the pressure for the lactating sows (Miller et al., 2012; Novotni-Dankó et al., 2015).

Although consumption of creep feed can lead to higher feed intake after weaning with concomitant greater growth performance (Bruininx et al., 2002; Pluske et al., 2007; Muns and Magowan, 2018), the application of creep feed still comes with variable feed intake, limited impact on the gut maturation during the pre-weaning period or the later life phases of piglets (Sulabo et al., 2010; Van den Brand et al., 2014). Meanwhile, the presence of maternal fecal semiochemicals in farrowing crates is also appealing to piglets (Aviles-Rosa et al., 2020), and gastrointestinal microbiota is primarily influenced by maternal and environmental factors (Lührmann et al., 2021; Nowland et al., 2021). Therefore, there is room to improve the early solid nutrition of piglets to activate their appetite and gut maturation.

In a semi-natural environment, piglets living outdoors already begin chewing straw and rooting during the second week of life (Schouten, 1985; Petersen, 1994). Our previous research on feral piglets showed spontaneous solid feeding intake from the first week of life, primarily leaves and stems, accounting for 83% of their stomach contents. This intake of fibrous plant matter was associated with a stronger stomach development (Van Hees et al., 2022). Compared to feral piglets, farm-raised piglets are usually only able to access creep feed formulated as being nutrient-dense and digestible. Therefore, the inclusion of fibrous content in creep feed is a promising avenue to explore, and it was reported to support the piglets’ behavior, including recognition memory, and frequent exploration and interaction with littermates (Clouard et al., 2018; Fleming et al., 2019). Meanwhile, in terms of physiology, a study by our group showed that the addition of dietary insoluble fiber in creep feed activated the colonic fermentation of short-chain fatty acids (SCFAs) and increased large intestinal size and fill, which strengthened the health of the digestive tract (Van Hees et al., 2019). These studies included the fiber sources as a ground ingredient mixed in the creep feed, which is obviously different from the intake of structure-rich plant parts in the wild. Grass hay is rarely fed to farmed pigs, but a common part of the natural diet of wild boar (Groot Bruinderink et al., 1994).

Because of the above, we wanted to mimic a part of the natural piglet diet by providing grass hay to suckling piglets. The objective of this study was to investigate whether a grass hay supplement would stimulate the appetite of piglets and foster development of a stronger gastrointestinal tract from an early age, enabling them to cope with weaning stress and achieve better performance in later phases.

## 2 Materials and methods

The housing and rearing of experimental animals were in compliance with European Union Directive 2010/63/EU. The protocols and procedures of this study were approved by the Ethics Committee of Flanders Research Institute for Agriculture, Fisheries and Food (ILVO), with application number 2022/420, and all animal experiments complied with the ARRIVE guidelines 2.0.

### 2.1 Animal housing and management

Fifteen sows (TN 70; average parity 3.9, from 1 to 8) were involved in this study. They were housed individually in the farrowing room of the research farm (ILVO, Melle, Belgium) 7 days prior to the expected farrowing date. The farrowing crates for sows were equipped with slatted floor including a heat lamp, a drinking nipple, a jute sack, and a feed trough that avoided the piglets consuming sow feed. The environmental temperature was controlled at approximately 23°C, and lighting was provided between 7:00 and 16:00 and dimmed during the night. The sows’ diet was offered *ad libitum* and mainly based on wheat, barley, and maize containing 155 g of crude protein, 55 g of crude fiber, and 51 g of crude fat per kg during lactation. The day when most litters were born was defined as d0 of this experiment and the piglets were weighed immediately after birth and identified individually by ear tags individually. The litter size was standardized to 14.2 ± 1.0 piglets within 3 days after farrowing by cross-fostering within these 15 sows in terms of the available nipples of sows and birth weight of piglets. Surgical castration and teeth clipping were not applied. The sleeping area for the piglets was heated by infrared light and floor heating was provided from birth to day 14. All piglets were offered creep feed *ad libitum* from day 2 onwards and another water nipple for the piglets fixed to the farrowing crate is also available. Weaning took place at day 28. Then, the piglets were moved to the nursery units on day 63. The arrangement for nursery phase is described below.

### 2.2 Experimental design and treatments

Sows were allocated to one of two treatments based on balanced parities. Seven litters were designated as the control group (CON), and piglets in the control group received the common concentrated creep feed (**Table 1**) in a round feeder from day 2 to weaning (day 28). In the treated group (GH), piglets received the same creep feed, but additionally, chopped grass hay (particle size: 6.0 ± 1.0 cm; analyzed nutritional components: dry matter 93.1%, crude protein 7.7%, crude fat 1.0%, crude ash 7.1%, NDF 53.1%, ADF 26.7%) in a separate feeder until weaning. The positions of creep feeders were identical across all litters, and grass hay feeders in the GH group were positioned equivalently and adjacent to the creep feeder. All piglets had ad libitum access to creep feed or grass hay and water. The weight of all diets in the feeders was recorded and leftovers were replaced by fresh material and collected by a vacuum cleaner on a litter basis every morning. As weaning approached, the suckling piglets were gradually transitioned from creep feed to weaner diets starting at 7 days prior to weaning.

**Table 1:** Composition (ingredients, nutrients) of creep feed (% as-fed basis) fed from day 2 to day 28 (day of weaning).

Ingredient	Creep feed
Composition, % as-fed basis	
Barley	35.00
Maize	15.00
Wheat	12.00
Toasted soy beans	10.00
Premix based on whey powder^1^	9.00
Soybean meal	7.59
Beet molasses	3.01
Potato protein	2.00
Wheat gluten	2.00
Soy oil	0.99
Mono calcium phosphate	0.82
Limestone	0.79
L-lysine HCl	0.58
Salt	0.46
L-threonine	0.24
DL-methionine	0.22
L-valine	0.13
L-tryptophan	0.08
Phytase (Ronozyme^®^)^2^	0.10
**Net energy, MJ/kg**	9.950
Nutrients, % as-fed basis	
Crude protein	17.90
Crude fiber	2.00
Crude fat	8.70
Ash	6.50
Available phosphorus	0.50
Calcium	0.60
Lysine	1.40
Methionine	0.50

^1^The premix contained 80% dairy product and 20% vitamin and mineral premix (i.e., per kilogram total feed, vitamin A, 15,000 IU; vitamin D3, 2,000 IU; vitamin E, 100 mg; vitamin K, 10 mg; vitamin B1, 3 mg; vitamin B2, 10 mg; vitamin B5, 25 mg; vitamin B6, 6 mg; vitamin B12, 0.04 mg; vitamin C, 100 mg; vitamin PP, 35 mg; choline, 416 mg; folic acid, 3.5 mg; biotin, 0.3 mg; Ca, 340 mg; P, 504 mg; Mg, 168 mg; Na, 591 mg; Cl, 995 mg; K, 2,017 mg; S, 205 mg; Fe, 100 mg; Cu, 140 mg; Mn, 60 mg; Zn, 100 mg; I, 2 mg; Se, 0.4 mg).

^2^Ronozyme Hiophos, 1–500 and 500–1,000 (1:1) phytase units/kg.

Upon weaning, piglets were moved to the nursery unit. Six piglets from the same litter with median weight and, as far as possible, balanced gender, were selected and assigned to one of the nursery pens (1.0 m × 2.0 m), resulting in 15 pens in total. All selected piglets only received common commercial nursery feed based on barley, corn, and wheat (consisting of 17.5% crude protein, 3.6% crude fiber, 5.0% crude fat, and 6.6% ash) after weaning and the remaining piglets were moved to other nursery pens to follow normal breeding procedures of this farm. The selected piglets had *ad libitum* access to nursery feed and drinking water and were monitored until 9 weeks of age (day 63).

### 2.3 Sampling and measurement

The individual body weight of piglets was measured at birth, on day 14, at weaning (day 28), and on day 42 and day 63. The disappearance of creep feed and grass hay was daily recorded to calculate the feed intake per litter. One day before weaning, one piglet per litter with a median body weight was selected for euthanasia by intra-cardiac injection with 30% barbiturate pentobarbital injection (Release, WDT Co. Germany) after sedation with 0.22 mL/kg Zoletil (Covertrus Co. USA). A midline laparotomy was performed to excise and separate each section of gastrointestinal tract. The full and emptied stomach, liver, and spleen were weighed. The small intestine (SI) and large intestine (LI) were ligated at their respective junctions, and their lengths were recorded on a dissection table while in a relaxed state. The SI and LI were then weighed both with and without contents. Additionally, 3-cm^2^ tissue samples at the same middle position from duodenum, jejunum, ileum, and mid-colon were washed with phosphate-buffered saline (PBS) and collected into a 4% formaldehyde solution used for microscopic examination. The representative and homogenized digesta from cecum and mid-colon were collected on dry ice first and subsequently transferred and stored to −80°C until analysis.

### 2.4 Luminal contents’ metabolic profile

The SCFAs of digesta were quantified according to the approach used by Gadeyne et al. (2016). Five milliliters of 10% formic acid containing the internal standard (1 mg of 2-ethyl butanoic acid) was added to 1 mL content, and after 15 min of centrifugation (22,000 *g* at 4°C), the supernatant was filtered and an aliquot was transferred into a 1.5-mL glass vial. The SCFAs were measured by gas chromatography (HP 7890A, Agilent Technologies, Diegem, Belgium), equipped with a flame ionization detector and a Supelco Nukol capillary column (30 m × 0.25 mm × 0.25 µm, Sigma-Aldrich, Diegem, Belgium).

### 2.5 Intestinal morphometry evaluation

Five-micrometer-thick sections of intestinal organs were cut from paraffin-embedded blocks (Sadeghipour and Babaheidarian, 2019) and then stained with hematoxylin–eosin (H&E) for light-microscopic examination (Leica DM LB2 with microscope imaging software from Leica Microsystems). For each slice, 10 intact villi or crypts (in mid-colon) of each slice were randomly selected and each sample had four slices serving as the replicates. The villus height and the depth of crypt adjacent to the selected villi were determined. The ratios between villus height and crypt depth were also calculated.

### 2.5 Mid-colon microbiota community analysis

To analyze the microbiota community, the representative luminal samples from mid-colon were taken and stored on dry ice and transferred to −80°C immediately. The microbial DNA in samples were extracted using PowerSoilPro (QiaGen, Germany) with a beat-beating step of 5 × 4,000 rpm for 15 s with 45-s intervals using a PowerLyzer instrument (QiaGen). Then, the DNA was eluted in 50 µL of elution buffer. Ten microliters of genomic DNA extract was sent out to LGC genomics GmbH (Berlin, Germany) where the 16S rRNA gene V3–V4 hypervariable region was amplified. The PCR mix included 1 µL of DNA extract, 15 pmol of both the forward primer 341F 5’-NNNNNNNNNTCCTACGGGNGGCWGCAG and reverse primer 785R 5’-NNNNNNNNNNTGACTACHVGGGTATCTAAKCC (Klindworth et al., 2013) in 20 µL volume of MyTaq buffer containing 1.5 units of MyTaq DNA polymerase (Bioline) and 2 µL of BioStabII PCR Enhancer (Sigma). The reaction conditions were carried out for 30 cycles using the following parameters: 2 min at 96°C for predenaturation; 96°C for 15 s, 50°C for 30 s, and 70°C for 90 s. The DNA concentration of amplicons of interest was determined by gel electrophoresis. The amplicon pools were purified with one volume AMPure XP beads (Agencourt) to remove primer dimer and other small mispriming products, followed by an additional purification on MinElute columns (Qiagen, Germany). Illumina libraries were pooled and size-selected by preparative gel electrophoresis. Sequencing was performed on an Illumina MiSeq using v3 Chemistry (Illumina).

### 2.6 Statistical analysis

Statistical analysis was performed using a general linear model to evaluate the effect of dietary treatment on growth performance and GIT characteristics with the litter at suckling phase and pen at post-weaning phase as the experimental unit by SPSS version 27.0 software (IBM SPSS Inc., USA). A covariate was retained when its *p*-value was less than 0.200 and not affected by treatment. Consequently, the birth weight to the growth performance and body weight at dissection were included as the covariates. For all analyses, we applied the Tukey–Kramer correction for *post-hoc* multiple comparison. Differences were considered significant if *p* < 0.05 while a tendency was considered when 0.05 < *p* < 0.100.

The DADA2 R package was used to process the amplicon sequence data according to the pipeline tutorial (Callahan et al., 2016). Finally, the amplicon sequence variant (ASV) table obtained after chimera removal was used for taxonomy assignment using the Naive Bayesian Classifier and the DADA2 formatted Silva v138 (Quast et al., 2013).

## 3 Results

Three piglets died within 3 days after treatment due to crushing and they were excluded from the results. Generally, according to daily monitoring, the animals in this study exhibited good clinical health throughout the experiment.

### 3.1 Growth performance

There were no significant differences in the results of weight gain between the groups until weaning, but when we categorized the piglets into three levels based on their birth weight within the same litter, the piglets with low birth weight numerically showed the greatest difference (CON vs. GH: 5.73 kg vs. 6.38 kg) in weight gain from birth to weaning compared to other levels (**Table 2**). The feed intake of creep feed showed high variation among litters. The piglets in GH displayed a consistently higher feed intake than those in CON over the 4-week lactation period, and a significant difference was observed in the second week (14.4 vs. 21.9 g/piglet, *p* < 0.05). Throughout the entire lactation period, the average grass hay feed intake of piglets exceeded 57 g of grass hay in total. After weaning, no significant differences were observed between the two treatments in weight gain and feed intake (*p* > 0.05).

**Table 2:** Technical performance of piglets fed a common creep feed with or without access to chopped grass hay from birth to 9 weeks of age.

Item	CON	GH	SEM^2^	*p-*Diet
Litters	7	8	–	–
Litter size (after standardization)	14.1	14.3	0.5	0.847
Birth weight, kg	1.49	1.58	0.16	0.385
Body weight day 14, kg	4.09	4.34	0.18	0.158
Body weight at weaning, kg	7.9	8.4	0.5	0.335
Average daily gain during lactation, g/day^1^	245	249	30	0.438
Weight gain with low birth weight during lactation, kg	5.7	6.4	1.6	0.166
Weight gain with median birth weight during lactation, kg	6.5	6.6	1.6	0.827
Weight gain with high birth weight during lactation, kg	6.8	7.3	1.6	0.258
Creep feed intake week 1, g/piglet	1.9	1.9	1.3	0.927
Creep feed intake week 2, g/piglet	14^b^	22^a^	8	0.049
Creep feed intake week 3, g/piglet	53	57	18	0.501
Creep feed intake week 4, g/piglet	116	133	33	0.221
Grass hay feed intake during lactation, g/piglet	–	57	3	–
Amount of selected piglets in nursery phase	54	56		
Average daily gain during the 5 weeks post weaning, g/day^1^	343	347	12	0.761
Feed intake during 5 weeks post weaning, g/piglet/day	539	553	72	0.728

CON, control group; GH, grass hay group; same as below.

^a,b^Mean values within a row with different superscripts differ significantly (p < 0.05).

^1^Birth weight as covariate.

^2^Pooled standard error of the mean.

### 3.2 Gastrointestinal tract morphometry

The body weight of selected piglets for necropsy was not significantly different, despite a numerical difference of 9% between two groups (**Table 3**, *p* > 0.05). During the necropsy, it was observed that both the absolute and relative weight of the stomach (both full and empty), liver, and spleen were not affected by the presence of grass hay (*p* > 0.05). However, grass hay increased the weight of emptied small intestine and large intestine significantly (280 vs. 228 g, 88.8 vs. 79.3 g, *p* = 0.008 and *p* = 0.033, respectively). Additionally, the large intestine was also found to be longer in GH than CON (*p* = 0.004). Nevertheless, there is no statistical difference in the relative weight of SI and LI to body weight (*p* > 0.05).

**Table 3:** The gastrointestinal tract morphometrics of piglets fed a common creep feed with or without access to chopped grass hay during the suckling phase.

Item	CON	GH	SEM^2^	*p*-Diet
Body weight at necropsy, kg	7.6	8.4	1.2	0.248
Full stomach weight, g^1^	170	193	33	0.642
Empty stomach weight, g^1^	52	55	2	0.415
Spleen weight, g^1^	45	49	2	0.241
Liver weight, g^1^	210	215	5	0.516
Small intestine length, cm^1^	881	942	35	0.253
Full SI weight, g^1^	285	350	31	0.181
Emptied SI weight, g^1^	228^b^	280^a^	11	0.008
Large intestine length, cm^1^	150^b^	164^a^	2	0.004
Full LI weight, g^1^	122	134	8	0.341
Emptied LI weight, g^1^	79^b^	89^a^	3	0.033
Weight relative to body weight,%				
Stomach	6.9	6.7	0.4	0.793
Spleen	5.5	6.1	0.3	0.159
Liver	26	27	2	0.354
Small intestine	29	34	2	0.093
Large intestine	10.3	10.9	0.5	0.388

CON, control group; GH, grass hay group; same as below (n = 7; n = 8).

SI, small intestine; LI, large intestine.

^a,b^Mean values within a row with different superscripts differ significantly (p < 0.05).

^1^Body weight at autopsy as covariate.

^2^Pooled standard error of the mean.

### 3.3 Microscopic morphometry of intestine

**Figure 1:**
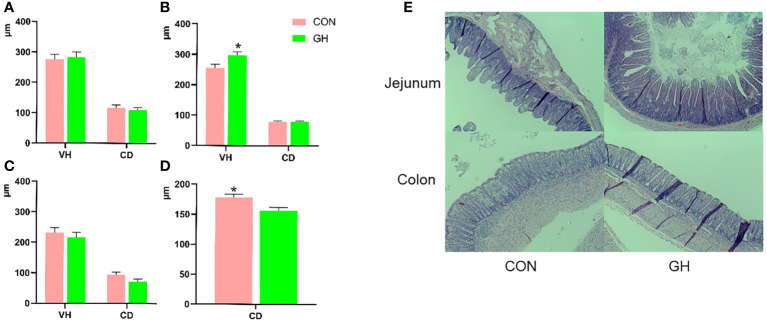
The villus height and crypt depth in intestine of piglets fed a common creep feed with or without grass hay during suckling phase. CON: control group, GH: grass hay group. **(A–D)** The comparison of villus height and crypt depth between two treatments. **(A)** Duodenum, **(B)** jejunum, **(C)** ileum, **(D)** mid-colon. **(E)** The typical pictures of microscopic morphology in jejunum and mid-colon. Body weight at necropsy as covariate; VH, villus height; CD, crypt depth. Columns marked with * at the top indicate significant difference (*p* < 0.05).

In the small intestine, the villus height and crypt depth in duodenum and ileum were not influenced by the treatments (**Figure 1**, *p* > 0.05). However, the average ratio between villus height and crypt depth of the ileum was higher in GH compared to CON (**Table 4**, *p* < 0.05). Furthermore, the villus height of jejunum was 40 µm longer in GH than CON (*p* < 0.05). In the large intestine, the crypt depth in CON was larger than in GH at weaning (*p* < 0.05).

**Table 4:** The villus height:crypt depth ratios in the respective small intestinal sections of piglets fed a common creep feed with or without access to chopped grass hay during the suckling phase.

Sections	CON	GH	SEM^2^	*p*-Diet
Duodenum	2.40	2.67	0.18	0.371
Jejunum	3.34	3.76	0.55	0.193
Ileum	2.54^b^	2.94^a^	0.32	0.049

^a,b^Mean values within a row with different superscripts differ significantly (p < 0.05).

^2^Pooled standard error of the mean.

### 3.4 Short-chain fatty acid profile in cecal and colonic content

**Figure 2:**
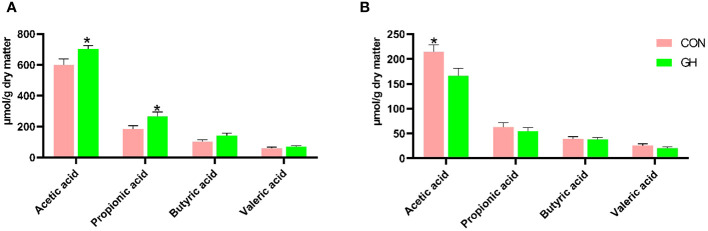
The short-chain fatty acid (SCFA) profile in cecal and colonic content of piglets fed a common creep feed with or without access to chopped grass hay during suckling phase. CON, control group; GH, grass hay group. **(A)** SCFA profile in cecal content. **(B)** SCFA profile in colonic content. Columns marked with * at the top indicate significant difference (*p* < 0.05). The error bars presented in the figure denote SEM.

In the cecum, grass hay increased the concentrations of acetic acid and propionic acid (**Figure 2**, 600 vs. 702 µmol/g dry matter for acetic acid, 185 vs. 255 µmol/g dry matter for propionic acid, *p* < 0.05). Additionally, the total SCFA concentration in the cecum of piglets in GH was also higher than CON, with values of 948 vs. 1,179 µmol/g dry matter (data not shown in **Figure 2**, *p* = 0.025). In contrast, in the mid-colon, the acetic acid concentration in GH was reduced (*p* < 0.05), and the total SCFA concentration in CON was numerically higher than GH (341 vs. 278 µmol/g dry matter).

### 3.5 Colonic microbiota community

**Figure 3:**
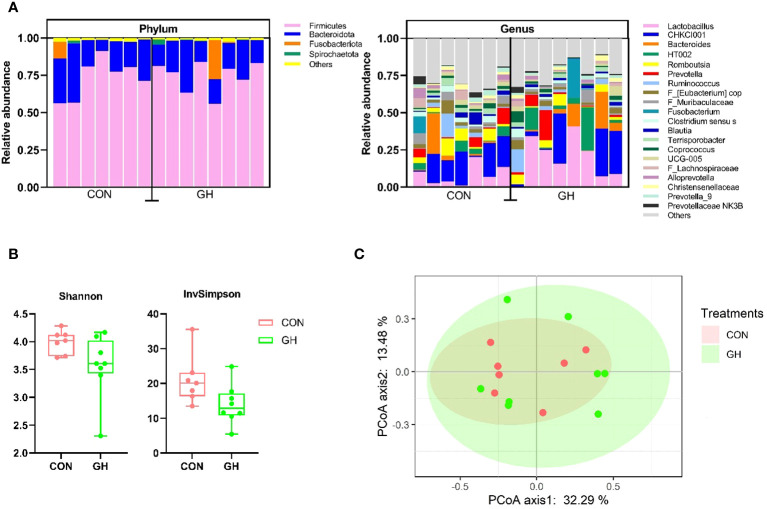
The microbiota community analysis in the mid-colon of piglets fed a common creep feed with or without access to chopped grass hay during suckling phase. CON, control group; GH, grass hay group. **(A)** Relative abundance of the 5 most abundant phylum and 20 most abundant genera in the mid-colon. **(B)** Diversity metrics, alpha-diversity in Shannon index, and inverse Simpson (InvSimpson) index. **(C)** Principal coordinates analysis (PcoA) of Bray–Curtis beta-diversity.

Within the relative proportions of ASV, the Firmicutes and Bacteroidota phyla collectively accounted for more than 95% of entire community, with Fusobacteriota and Spirochaetota following closely in the phylum level (**Figure 3A**). At the genus level, microbiota composition exhibited a large variation, and we observed that *Lactobacillus*, *CHKCI001*, and *Bacteroides* were the most three dominant genera across all samples. Furthermore, there was a trend that the average *Lactobacillus* proportion in GH was higher than with CON, accounting to 19.6% vs. 8.0% (*p* = 0.080). The supplement of grass hay did not affect the alpha-diversity at the ASV level, as indicated by the Shannon index comparison (**Figure 3C**, *p* > 0.05). However, the InvSimpson index tended to decrease in the GH group compared to CON (**Figure 3B**) (*p* = 0.051). The principal coordinates analysis (PCoA) on the microbial compositions of individuals showed no distinct clusters separating the two groups responsive to the treatment.

## 4 Discussion

This study investigated the impact of a separate provision of grass hay on supporting suckling piglets development. Our findings showed that the inclusion of a separate feeder containing chopped grass hay in the farrowing crate modified the metabolic profile and growth of the gastrointestinal tract. Fiber enrichment has been extensively tested in pig feeding due to its potential benefits, and selective inclusion of fiber was shown to promote GIT development and modify gut microbiome and immune status in finishing pigs and sows feeding (Jin et al., 1994; Mcglone and Fullwood, 2001; Claus et al., 2007; Heinritz et al., 2016). Unlike other feeding phases, appetite to solid feed of piglets in the suckling phase is markedly variable and unpredictable not only between litters but also within the same litter, even when providing what is considered a palatable formula (Pajor et al., 1991; Sulabo et al., 2010; Middelkoop et al., 2019a).

Therefore, the consistent increase in creep feed intake upon grass hay provision, is an important finding for practice. Other studies on inclusion of fiber in creep feed so far only showed numerical changes in creep feed intake (Hanczakowska et al., 2008; Van Hees et al., 2019), suggesting that the physical form of the grass hay in addition to its chemical traits may be important to affect voluntary solid feed intake in young piglets. Other types of insoluble fiber, such as wheat bran, have been observed to speed up the gastric mobility. This acceleration plays a crucial role in the gut–brain axis, regulating appetite signals to activate the feed intake and behaviors, which might contribute to the result in the present study (Benini et al., 1995; Inui et al., 2004; Li et al., 2023). Furthermore, it also might be interesting to investigate if the impact of feeding hay depends on the birth weight of piglets in a study involving more litters. The presence of extra feeders with diverse feed resources in the farrowing crate is capable of stimulating the suckling piglets’ exploring and foraging behaviors (Middelkoop et al., 2019b). This, in turn, might lead to a reduction in the competition among piglets for teats access, as it is known that piglets with heavier birth weight are known to consume more milk per suckling event than their lighter littermates (Campbell and Dunkin, 1982). However, some other previous studies demonstrated that the supplementation with dietary fiber could suppress the appetite (De Leeuw et al., 2008; Jarrett and Ashworth, 2018). It may be attributed to the fact that fermentation of SCFAs in the colon can stimulate the secretion of gut–brain peptides and mediate the appetite signals via the vagus nerve (Bolognini et al., 2021). Whether this pathway is primarily activated via the fermentation in colon rather than other sections of large intestine needs to be fully elucidated, and non-pronounced change of colonic microbiome occurred in this study, playing a key role in regulating host’s appetite, might also erase the decreased feed intake. Therefore, the effects seen in the GH group may go beyond the direct impact of fiber consumption.

The weight of visceral organs can reflect the piglet’s maturation process (Elefson et al., 2021), but the grass hay provision induced no effect on stomach, liver, and spleen weight. Len et al. (2009) studied fiber levels and sources in weaned piglets and also found no effect on liver weight, yet a higher stomach weight after 33 days of feeding. Studies on the effect of fiber on spleen weight are limited, but in general, the development of spleen and liver remains closely related to the body weight growth from birth to weaning (Lanferdini et al., 2018; Elefson et al., 2021). It is worth noting that a sufficient duration of exposure to the diet might also play a role in its effect. For instance, pigs provided with high-fiber diet for an extended period had heavier weights of liver and kidney weights compared to those on a lower fiber diet, but this effect was not as pronounced with a shorter feeding duration (Pond et al., 1988; Anugwa et al., 1989). Given the fairly short length of the suckling phase combined with the low intake in commercial farming, this aspect is hard to cope with. The response on stomach weight to insoluble fiber appears to be faster than other organs due to the bulking effect in weaned piglets (Rijnen et al., 2001; Len et al., 2009), although—as in our study—supplementing suckling piglets with cellulose and other fibers in creep feed did not alter the empty stomach weight (Van Hees et al., 2019; Choudhury et al., 2021).

Growing pigs fed high-fiber diets typically exhibit a larger gastrointestinal tract than those fed a common diet (Jørgensen et al., 1996; Freire et al., 2003; Pluske et al., 2003). We here demonstrated that this also occurs in suckling piglets when fed grass hay. This enlargement might be attributed to water-retentive and “bulking” properties of (insoluble) fiber (Tungland and Meyer, 2002; Dhingra et al., 2012), as observed in previous studies applying cellulose and oat hulls in creep feed (Van Hees et al., 2019). The absence of a significant difference in relative weight of small and large intestine might also suggest that the increased weight of intestine may not be sufficient to influence the overall carcass weight. Not only the macroscopic anatomy but also the microscopic architecture was affected, mainly shown by an increased ileal villus:crypt ratio, likely resulting from increased digestion of nutrients in the jejunum (Nigam et al., 2019). This observation may therefore reflect increased proximal nutrient digestion and absorption, rendering less challenge of undigested matter to the distal intestinal sections, hence explaining its higher villus:crypt ratio. Some of the changes in the villus:crypt ratio may originate from a response to intestinal injury induced by various agents, such as digestive pressure, promoting crypt hyperplasia (Da Cunha Ferreira et al., 1990; Potten, 1990; Pizarro et al., 2000).

A well-developed large intestine is essential for piglets absorbing fluid, electrolytes, and remaining nutrients, including SCFA, from the proximal sections efficiently. The physical barrier of the intestine also helps against microbial invasion (Williams et al., 2001; Xu and Cranwell, 2003). The 12% heavier and 10% longer large intestine due to grass hay thus is an interesting trait. Moreover, it coincides with increased production of SCFA, generating additional fuel for large intestinal enterocytes and cell proliferation (Scheppach, 1994). Further study is needed to identify the cause of the more proximal shift in fermentation site due to grass hay, but it is well-studied that cellulose-rich material on itself is poorly fermentable in pigs (Bachmann et al., 2021), especially young individuals. Hence, the likely explanation for the fermentation shift is an effect of the grass hay on the passage of the fermentable fractions of the ingested creep feed and milk oligosaccharides.

Abimosleh et al. (2012) have reported that induced colitis was accompanied by deeper crypt depth in rats, and imbalanced cell proliferation is also a risk factor of neoplasia (Deschner and Maskens, 1982). However, one trait such as crypt depth within a complex population of intestinal cells may not represent the entire function of the LI (Snippert, 2016). While this perspective is insightful regarding the current study, further investigation is required for confirming the association between the structural characteristics of LI and its optimal functioning, such as cell proliferation, nutrient absorption, and other relevant factors.

Despite marked changes in intestinal anatomy and morphology, consumption of grass hay did not exert major changes in colonic microbiota. Plenty of studies have reported the effect of fiber on the microbiota on weaning or fattening pigs (Kraler et al., 2016; Onarman Umu et al., 2018; Fouhse et al., 2019). Unlike older pigs, observing changes in microbiota composition in the suckling piglet intestine is challenging due to various factors such as creep feed patterns, the genital tract and feces of sow influence, genetic interactions, and pen environment, all of which contribute to the complex microbiota community (Kubasova et al., 2017; Chen et al., 2018). The trend of a lower alpha-diversity with GH in the colon may relate to the proximal shift of fermentation discussed higher. At the same time, the abrasive function of insoluble fiber may slough the epithelial mucus together with the microbes (Molist et al., 2009; Chen et al., 2013). The trend of increased abundance of *Lactobacillus* in the colonic microbiota when fed grass hay is in line with observations in rats and humans fed insoluble fiber (Gibson et al., 1995; Zhong et al., 2015), but in general, it is surprising that the microbiome was hardly affected by the grass hay intake.

Although there are still many lingering questions regarding long-term effects, feeding grass hay to suckling piglets shows promise and warrants further exploration in practical settings. Larger-scale studies will need to clarify if performance differences during suckling as well as throughout the entire fattening phase will appear.

## 5 Conclusion

Piglets readily consume grass hay in the pre-weaning period. Access to chopped grass hay during the suckling period in a separate feeder stimulated the feed intake of creep feed and promoted intestinal growth. Moreover, the consumption of grass hay exerted a more proximal shift in intestinal fermentation without prominent changes in microbial communities.

## Data availability statement

The raw data supporting the conclusions of this article will be made available by the authors, without undue reservation. The sequencing data are available in the NCBI Sequence Read Archive (SRA) under accession: PRJNA1043179.

## Ethics statement

The animal studies were approved by Ethics Committee of Flanders Research Institute for Agriculture, Fisheries and Food (ILVO), with application number 2022/420 and all animal experiments complied with the ARRIVE guidelines 2.0. The studies were conducted in accordance with the local legislation and institutional requirements. Written informed consent was obtained from the owners for the participation of their animals in this study.

## Author contributions

RY: Conceptualization, Data curation, Formal Analysis, Investigation, Methodology, Writing – original draft. AC: Conceptualization, Data curation, Formal Analysis, Investigation, Writing – review & editing. HH: Methodology, Writing – review & editing. KC: Methodology, Writing – review & editing. AM: Methodology, Writing – review & editing. MA: Methodology, Writing – review & editing. DM: Methodology, Supervision, Writing – review & editing. GJ: Conceptualization, Funding acquisition, Methodology, Project administration, Supervision, Writing – review & editing.

## References

[B1] Abimosleh S. M. Lindsay R. J. Butler R. N. Cummins A. G. Howarth G. S. (2012). Emu oil increases colonic crypt depth in a rat model of ulcerative colitis. Digestive Dis. Sci. 57, 887–896. doi: 10.1007/s10620-011-1979-1 22147247

[B2] Anugwa F. O. Varel V. H. Dickson J. S. Pond W. G. Krook L. P. (1989). Effects of dietary fiber and protein concentration on growth, feed efficiency, visceral organ weights and large intestine microbial populations of swine. J. Nutr. 119, 879–886. doi: 10.1093/jn/119.6.879 2545846

[B3] Aviles-Rosa E. O. Surowiec K. Mcglone J. (2020). Identification of Faecal Maternal Semiochemicals in Swine (Sus scrofa) and their Effects on Weaned Piglets. Sci. Rep. 10, 5349. doi: 10.1038/s41598-020-62280-9 32210329 PMC7093430

[B4] Bachmann M. Michel S. Greef J. M. Zeyner A. (2021). Fermentation characteristics and *in vitro* digestibility of fibers and fiber-rich byproducts used for the feeding of pigs. Anim. (Basel) 11, 341. doi: 10.3390/ani11020341 PMC7911969 33572852

[B5] Benini L. Castellani G. Brighenti F. Heaton K. W. Brentegani M. T. Casiraghi M. C.. (1995). Gastric emptying of a solid meal is accelerated by the removal of dietary fibre naturally present in food. Gut 36, 825–830. doi: 10.1136/gut.36.6.825 7615267 PMC1382616

[B6] Bolognini D. Dedeo D. Milligan G. (2021). Metabolic and inflammatory functions of short-chain fatty acid receptors. Curr. Opin. endocrine Metab. Res. 16, 1–9. doi: 10.1016/j.coemr.2020.06.005 32835130 PMC7332907

[B7] Bruininx E. M. Binnendijk G. P. van der Peet-Schwering C. M. Schrama J. W. Den Hartog L. A. Everts H.. (2002). Effect of creep feed consumption on individual feed intake characteristics and performance of group-housed weanling pigs. J. Anim. Sci. 80, 1413–1418. doi: 10.2527/2002.8061413x 12078720

[B8] Callahan B. J. Mcmurdie P. J. Rosen M. J. Han A. W. Johnson A. J. Holmes S. P. (2016). DADA2: High-resolution sample inference from Illumina amplicon data. Nat. Methods 13, 581–583. doi: 10.1038/nmeth.3869 27214047 PMC4927377

[B9] Campbell R. G. Dunkin A. C. (1982). The effect of birth weight on the estimated milk intake, growth and body composition of sow-reared piglets. Anim. Production 35, 193–197. doi: 10.1017/S0003356100027355

[B10] Chen H. Mao X. He J. Yu B. Huang Z. Yu J.. (2013). Dietary fibre affects intestinal mucosal barrier function and regulates intestinal bacteria in weaning piglets. Br. J. Nutr. 110, 1837–1848. doi: 10.1017/S0007114513001293 23656640

[B11] Chen X. Xu J. Ren E. Su Y. Zhu W. (2018). Co-occurrence of early gut colonization in neonatal piglets with microbiota in the maternal and surrounding delivery environments. Anaerobe 49, 30–40. doi: 10.1016/j.anaerobe.2017.12.002 29223548

[B12] Choudhury R. Middelkoop A. De Souza J. G. Van Veen L. A. Gerrits W. J. J. Kemp B.. (2021). Impact of early-life feeding on local intestinal microbiota and digestive system development in piglets. Sci. Rep. 11, 4213. doi: 10.1038/s41598-021-83756-2 33603087 PMC7892833

[B13] Claus R. Günthner D. Letzguss H. (2007). Effects of feeding fat-coated butyrate on mucosal morphology and function in the small intestine of the pig. J. Anim. Physiol. Anim. Nutr. (Berl) 91, 312–318. doi: 10.1111/j.1439-0396.2006.00655.x 17615002

[B14] Clouard C. Stokvis L. Bolhuis J. E. Van Hees H. M. J. (2018). Short communication: insoluble fibres in supplemental pre-weaning diets affect behaviour of suckling piglets. Animal 12, 329–333. doi: 10.1017/S1751731117001501 28701236

[B15] Da Cunha Ferreira R. Forsyth L. E. Richman P. I. Wells C. Spencer J. Macdonald T. T. (1990). Changes in the rate of crypt epithelial cell proliferation and mucosal morphology induced by a T-cell-mediated response in human small intestine. Gastroenterology 98, 1255–1263. doi: 10.1016/0016-5085(90)90342-X 2138987

[B16] De Leeuw J. A. Bolhuis J. E. Bosch G. Gerrits W. J. (2008). Effects of dietary fibre on behaviour and satiety in pigs. Proc. Nutr. Soc. 67, 334–342. doi: 10.1017/S002966510800863X 18715518

[B17] Deschner E. E. Maskens A. P. J. C. (1982). Significance of the labeling index and labeling distribution as kinetic parameters in colorectal mucosa of cancer patients and DMH treated animals. Cancer 50, 1136–1141. doi: 10.1002/1097-0142(19820915)50:6<1136::AID-CNCR2820500617>3.0.CO;2-A 7104954

[B18] Dhingra D. Michael M. Rajput H. Patil R. T. (2012). Dietary fibre in foods: a review. J. Food Sci. Technol. 49, 255–266. doi: 10.1007/s13197-011-0365-5 23729846 PMC3614039

[B19] Elefson S. K. Lu N. Chevalier T. Dierking S. Wang D. Monegue H. J.. (2021). Assessment of visceral organ growth in pigs from birth through 150 kg. J. Anim. Sci. 99, skab249. doi: 10.1093/jas/skab249 34435641 PMC8438542

[B20] Fleming S. A. Monaikul S. Patsavas A. J. Waworuntu R. V. Berg B. M. Dilger R. N. (2019). Dietary polydextrose and galactooligosaccharide increase exploratory behavior, improve recognition memory, and alter neurochemistry in the young pig. Nutr. Neurosci. 22, 499–512. doi: 10.1080/1028415X.2017.1415280 29251222

[B21] Fouhse J. M. Dawson K. Graugnard D. Dyck M. Willing B. P. (2019). Dietary supplementation of weaned piglets with a yeast-derived mannan-rich fraction modulates cecal microbial profiles, jejunal morphology and gene expression. Animal 13, 1591–1598. doi: 10.1017/S1751731118003361 30614425

[B22] Freire J. P. B. Dias R. I. M. Cunha L. F. Aumaitre A. (2003). The effect of genotype and dietary fibre level on the caecal bacterial enzyme activity of young piglets: digestive consequences. Anim. Feed Sci. Technol. 106, 119–130. doi: 10.1016/S0377-8401(03)00003-8

[B23] Gadeyne F. De Ruyck K. Van Ranst G. De Neve N. Vlaeminck B. Fievez V. (2016). Effect of changes in lipid classes during wilting and ensiling of red clover using two silage additives on in *vitro* ruminal biohydrogenation. J. Agric. Sci. 154, 553–566. doi: 10.1017/S0021859615001203

[B24] Gibson G. R. Beatty E. R. Wang X. Cummings J. H. J. G. (1995). Selective stimulation of bifidobacteria in the human colon by oligofructose and inulin. Gastroenterology 108, 975–982. doi: 10.1016/0016-5085(95)90192-2 7698613

[B25] Groot Bruinderink G. W. T. A. Hazebroek E. van der Voot H. (1994). Diet and condition of wild boar, Sus scrofu scrofu, without supplementary feeding. J. Zoology 233, 631–648. doi: 10.1111/j.1469-7998.1994.tb05370.x

[B26] Hanczakowska E. Świ̧Tkiewicz M. Białecka A. (2008). Pure cellulose as a feed supplement for piglets. Medycyna Weterynaryjna 64, 45–48.

[B27] Heinritz S. N. Weiss E. Eklund M. Aumiller T. Heyer C. M. Messner S.. (2016). Impact of a high-fat or high-fiber diet on intestinal microbiota and metabolic markers in a pig model. Nutrients 8, 317. doi: 10.3390/nu8050317 27223303 PMC4882729

[B28] Inui A. Asakawa A. Y. Bowers C. Mantovani G. Laylano A. M. Meguid M.. (2004). Ghrelin, appetite, and gastric motility: the emerging role of the stomach as an endocrine organ. FASEB J. 18, 439–456. doi: 10.1096/fj.03-0641rev 15003990

[B29] Jarrett S. Ashworth C. J. (2018). The role of dietary fibre in pig production, with a particular emphasis on reproduction. J. Anim. Sci. Biotechnol. 9, 59. doi: 10.1186/s40104-018-0270-0 30128149 PMC6091159

[B30] Jin L. Reynolds L. P. Redmer D. A. Caton J. S. Crenshaw J. D. (1994). Effects of dietary fiber on intestinal growth, cell proliferation, and morphology in growing pigs. J. Anim. Sci. 72, 2270–2278. doi: 10.2527/1994.7292270x 7528192

[B31] Jørgensen H. Zhao X.-Q. Eggum B. O. (1996). The influence of dietary fibre and environmental temoperature on the development of the gastrointestinal tract, digestibility, degree of fermentation in the hind-gut and energy metabolism in pigs. Br. J. Nutr. 75, 365–378. doi: 10.1079/bjn19960140 8785211

[B32] Klindworth A. Pruesse E. Schweer T. Peplies J. Quast C. Horn M.. (2013). Evaluation of general 16S ribosomal RNA gene PCR primers for classical and next-generation sequencing-based diversity studies. Nucleic Acids Res. 41, e1. doi: 10.1093/nar/gks808 22933715 PMC3592464

[B33] Kraler M. Ghanbari M. Domig K. J. Schedle K. Kneifel W. (2016). The intestinal microbiota of piglets fed with wheat bran variants as characterised by 16S rRNA next-generation amplicon sequencing. Arch. Anim. Nutr. 70, 173–189. doi: 10.1080/1745039X.2016.1160534 27032029

[B34] Kubasova T. Davidova-Gerzova L. Merlot E. Medvecky M. Polansky O. Gardan-Salmon D.. (2017). Housing systems influence gut microbiota composition of sows but not of their piglets. PloS One 12, e0170051. doi: 10.1371/journal.pone.0170051 28085934 PMC5234784

[B35] Lallès J. P. Bosi P. Smidt H. Stokes C. R. (2007). Nutritional management of gut health in pigs around weaning. Proc. Nutr. Soc. 66, 260–268. doi: 10.1017/S0029665107005484 17466106

[B36] Lanferdini E. Andretta I. Fonseca L. S. Moreira R. H. R. Cantarelli V. S. Ferreira R. A.. (2018). Piglet birth weight, subsequent performance, carcass traits and pork quality: A meta-analytical study. Livestock Sci. 214, 175–179. doi: 10.1016/j.livsci.2018.05.019

[B37] Len N. T. Hong T. T. T. Ogle B. Lindberg J. E. (2009). Comparison of total tract digestibility, development of visceral organs and digestive tract of Mong cai and Yorkshire × Landrace piglets fed diets with different fibre sources. J. Anim. Physiol. Anim. Nutr. (Berl) 93, 181–191. doi: 10.1111/j.1439-0396.2007.00804.x 19320931

[B38] Li S. Liu M. Cao S. Liu B. Li D. Wang Z.. (2023). The mechanism of the gut-brain axis in regulating food intake. Nutrients 15, 3728. doi: 10.3390/nu15173728 37686760 PMC10490484

[B39] Lührmann A. Ovadenko K. Hellmich J. Sudendey C. Belik V. Zentek J.. (2021). Characterization of the fecal microbiota of sows and their offspring from German commercial pig farms. PloS One 16, e0256112. doi: 10.1371/journal.pone.0256112 34398927 PMC8367078

[B40] Mcglone J. J. Fullwood S. D. (2001). Behavior, reproduction, and immunity of crated pregnant gilts: effects of high dietary fiber and rearing environment. J. Anim. Sci. 79, 1466–1474. doi: 10.2527/2001.7961466x 11424683

[B41] Middelkoop A. Costermans N. Kemp B. Bolhuis J. E. (2019a). Feed intake of the sow and playful creep feeding of piglets influence piglet behaviour and performance before and after weaning. Sci. Rep. 9, 16140. doi: 10.1038/s41598-019-52530-w 31695101 PMC6834851

[B42] Middelkoop A. Van Marwijk M. A. Kemp B. Bolhuis J. E. (2019b). Pigs like it varied; feeding behavior and pre- and post-weaning performance of piglets exposed to dietary diversity and feed hidden in substrate during lactation. Front. Vet. Sci. 6, 408. doi: 10.3389/fvets.2019.00408 31803769 PMC6877737

[B43] Miller Y. J. Collins A. M. Smits R. J. Thomson P. C. Holyoake P. K. (2012). Providing supplemental milk to piglets preweaning improves the growth but not survival of gilt progeny compared with sow progeny. J. Anim. Sci. 90, 5078–5085. doi: 10.2527/jas.2011-4272 22829606

[B44] Molist F. De Segura A. G. Gasa J. Hermes R. Manzanilla E. Anguita M.. (2009). Effects of the insoluble and soluble dietary fibre on the physicochemical properties of digesta and the microbial activity in early weaned piglets. Anim. Feed Sci. 149, 346–353. doi: 10.1016/j.anifeedsci.2008.06.015

[B45] Muns R. Magowan E. J. J. O. A. S. (2018). The effect of creep feed intake and starter diet allowance on piglets’ gut structure and growth performance after weaning. J. Anim. Sci. 96, 3815–3823. doi: 10.1093/jas/sky239 29924319 PMC6127789

[B46] Nigam Y. Knight J. Williams N. J. N. T. (2019). Gastrointestinal tract 4: anatomy and role of the jejunum and ileum. Nursing Time 115, 41–44.

[B47] Novotni-Dankó G. Balogh P. Huzsvai L. Győri Z. J. A. A. B. (2015). Effect of feeding liquid milk supplement on litter performances and on sow back-fat thickness change during the suckling period. Arch. Anim. Breed. 58, 229–235. doi: 10.5194/aab-58-229-2015

[B48] Nowland T. L. Kirkwood R. N. Plush K. J. Barton M. D. Torok V. A. (2021). Exposure to maternal feces in lactation influences piglet enteric microbiota, growth, and survival preweaning. J. Anim. Sci. 99, skab170. doi: 10.1093/jas/skab170 34036347 PMC8259832

[B49] Onarman Umu Ö.C. Fauske A. K. Åkesson C. P. Pérez De Nanclares M. Sørby R. Press C. M.. (2018). Gut microbiota profiling in Norwegian weaner pigs reveals potentially beneficial effects of a high-fiber rapeseed diet. PloS One 13, e0209439. doi: 10.1371/journal.pone.0209439 30571797 PMC6301702

[B50] Pajor E. A. Fraser D. Kramer D. L. (1991). Consumption of solid food by suckling pigs: individual variation and relation to weight gain. Appl. Anim. Behav. Sci. 32, 139–155. doi: 10.1016/S0168-1591(05)80038-3

[B51] Petersen V. J. A. A. B. S. (1994). The development of feeding and investigatory behaviour in free-ranging domestic pigs during their first 18 weeks of life. Appl. Anim. Behav. Sci. 42, 87–98. doi: 10.1016/0168-1591(94)90149-X

[B52] Pizarro T. T. Arseneau K. O. Cominelli F. J. A. J. O. P.-G. Physiology L. (2000). XI. Novel mouse models to study pathogenic mechanisms of Crohn’s disease. Am. J. Physiol. Gastrointest. Liver Physiol. 278, G665–G669. doi: 10.1152/ajpgi.2000.278.5.G665 10801257

[B53] Pluske J. R. Kerton D. K. Cranwell P. D. Campbell R. G. Mullan B. P. King R. H.. (2003). Age, sex, and weight at weaning influence organ weight and gastrointestinal development of weanling pigs. J. Aust. J. Agric. Res. 54, 515–527. doi: 10.1071/AR02156

[B54] Pluske J. R. Kim J. C. Hansen C. F. Mullan B. P. Payne H. G. Hampson D. J.. (2007). Piglet growth before and after weaning in relation to a qualitative estimate of solid (creep) feed intake during lactation: a pilot study. Arch. Anim. Nutr. 61, 469–480. doi: 10.1080/17450390701664249 18069618

[B55] Pond W. G. Jung H. G. Varel V. H. (1988). Effect of dietary fiber on young adult genetically lean, obese and contemporary pigs: body weight, carcass measurements, organ weights and digesta content. J. Anim. Sci. 66, 699–706. doi: 10.2527/jas1988.663699x 2837444

[B56] Potten C. S. J. I. J. O. R. B. (1990). A comprehensive study of the radiobiological response of the murine (BDF1) small intestine. Int. J. Radiat. Biol. 58, 925–973. doi: 10.1080/09553009014552281 1978853

[B57] Quast C. Pruesse E. Yilmaz P. Gerken J. Schweer T. Yarza P.. (2013). The SILVA ribosomal RNA gene database project: improved data processing and web-based tools. Nucleic Acids Res. 41, D590–D596. doi: 10.1093/nar/gks1219 23193283 PMC3531112

[B58] Rijnen M. Dekker R. Bakker G. Verstegen M. Schrama J. (2001). “Effects of dietary fermentable carbohydrates on the empty weights of the gastrointestinal tract in growing pigs,” in Digestive physiology of pigs. Proceedings of the 8th Symposium, Swedish University of Agricultural Sciences, Uppsala, Sweden, 20-22 June 2000. 17–20 (CABI Publishing Wallingford UK).

[B59] Sadeghipour A. Babaheidarian P. (2019). Making formalin-fixed, paraffin embedded blocks. Methods Mol. Biol. 1897, 253–268. doi: 10.1007/978-1-4939-8935-5_22 30539450

[B60] Scheppach W. (1994). Effects of short chain fatty acids on gut morphology and function. Gut 35, S35–S38. doi: 10.1136/gut.35.1_Suppl.S35 8125387 PMC1378144

[B61] Schouten W. G. P. (1985). Rearing conditions and behaviour in pigs (Netherlands: Wageningen University and Research).

[B62] Snippert H. J. (2016). Colonic crypts: safe haven from microbial products. Cell 165, 1564–1566. doi: 10.1016/j.cell.2016.06.003 27315471

[B63] Sulabo R. C. Jacela J. Y. Tokach M. D. Dritz S. S. Goodband R. D. Derouchey J. M.. (2010). Effects of lactation feed intake and creep feeding on sow and piglet performance1. J. Anim. Sci. 88, 3145–3153. doi: 10.2527/jas.2009-2131 20495122

[B64] Summers K. L. Frey J. F. Ramsay T. G. Arfken A. M. J. J. O. A. S. (2019). The piglet mycobiome during the weaning transition: a pilot study. J. Anim. Sci. 97, 2889–2900. 31136650 10.1093/jas/skz182 PMC6606507

[B65] Tungland B. C. Meyer D. (2002). Nondigestible oligo- and polysaccharides (Dietary fiber): their physiology and role in human health and food. Compr. Rev. Food Sci. Food Saf. 1, 90–109. doi: 10.1093/jas/skz182 33451232

[B66] Van den Brand H. Wamsteeker D. Oostindjer M. Van Enckevort L. van der Poel A. Kemp B.. (2014). Effects of pellet diameter during and after lactation on feed intake of piglets pre-and postweaning. J. Anim. Sci. 92, 4145–4153. doi: 10.2527/jas.2014-7408 25185217

[B67] Van Hees H. M. J. Ballari S. A. Dieste-Pérez L. Carpinetti B. N. Janssens G. P. J. (2022). Diet and stomach characteristics of feral piglets (Sus scrofa): Implications for farmed piglets. J. Anim. Physiol. Anim. Nutr. (Berl). 107 (2), 529–540. doi: 10.1111/jpn.13726 35603976

[B68] Van Hees H. M. J. Davids M. Maes D. Millet S. Possemiers S. Den Hartog L. A.. (2019). Dietary fibre enrichment of supplemental feed modulates the development of the intestinal tract in suckling piglets. J. Anim. Sci. Biotechnol. 10, 83. doi: 10.1186/s40104-019-0386-x 31636904 PMC6794736

[B69] Williams B. A. Verstegen M. W. Tamminga S. (2001). Fermentation in the large intestine of single-stomached animals and its relationship to animal health. Nutr. Res. Rev. 14, 207–228. doi: 10.1079/NRR200127 19087424

[B70] Xu R. J. Cranwell P. D. (2003). The neonatal pig: gastrointestinal physiology and nutrition (England: Nottingham University Press).

[B71] Zhong Y. Nyman M. Fåk F. (2015). Modulation of gut microbiota in rats fed high-fat diets by processing whole-grain barley to barley malt. Mol. Nutr. Food Res. 59, 2066–2076. doi: 10.1002/mnfr.201500187 26184884

